# Pathology

**Published:** 1890-11-22

**Authors:** 


### THE LONDON POST-GRADUATE COURSE AT THE PADDINGTON INFIRMARY.

### PATHOLOGY.

Dr. Savill, in his demonstration, first described the case of a man who was 63 years of age, and a crossing sweeper by occupation. He had been in the infirmary three times. On the last occasion he was admitted on October 28, complaining of pain in the back and legs, together with some weakness. On November 1st he became paralysed in both legs, and this was followed by incontinence of urine. On examination, there was found to be complete flaccid paralysis, with anæsthesia of both legs, and some slight angular curvature of the back in the upper dorsal region. The temperature was slightly raised, and remained so till November 10, when it was noticed that the abdomen was very tympanitic. He died on November 14, with symptoms of peritonitis, without the vomiting, which is usually present in this disease. Post-mortem: the peritoneal cavity was found to be full of a turbid fluid. and the intestines were congested and adherent in places with bands of recent lymph. In the sigmoid flexure there was found a small patch of ulceration without any thickening ground, and from this a small perforation extended upwards and opened behind the sigmoid flexure. On examination of the spinal cord a constriction was found in the middle dorsal region, and transverse myelitis had been set up. On removing the cord a small nodule of vertebral substance was seen projecting into the cord. The vertebral substance was eroded by a growth, the intervertebral discs remaining normal. The aorta was very atheromatous, and about half-way between the arch and the diaphragm there was a large aneumysmal sac on the posterior wall. In the heart the left ventricle was hypertrophied, the organ weighed ten and a-half ounces, the valves were slightly roughened, and there were considerable pericardial adhesions. The lungs showed some congestion and œdema of their bases, espcially of the right and some emphysema of the margins. The kidneys were slightly congested but otherwise normal. Some cases of joint disease were next described—the first, a man who died from an aneurysm complicated with pneumonia. He had had gout for some eight years before his death, and on examining his affected joints, it was very clearly seen how the cartilages and not the synovial fringes are affected by the deposit of urate of soda. Gout is due to an excess of uric acid in the blood. If in any case of gout a drachm of the blood or of the serum and six minims of acetic acid are mixed in a watch glass which contains a thread of cotton and left for a few hours, the thread when examined under the microscope has crystals of uric acid attached to it. An excess of uric acid in cases of gout is accounted for as follows: 1. The uric acid tends to become deposited in the tissues because there is an excess of acidity in the blood. 2. On account of a failure on the part of the kidneys to eliminate the acid normally present in the blood, for in birds and reptiles uric acid accumulates in ths blood after tying the renal vein, but in these forms we must remember that the urine normally contains much more uric acid than in the case of mammals. 3. It is a metabolic phenomenon, that is to say, there is an increased formation of acid owing to altered function of the body, and that it is the liver which is primarily at fault. 4. That the nervous system is at fault (neuro-trophic influence). There is one thing which influences gout, and that is the regulation of diet, and this is able not only to prevent the excessive formation of uric acid, but also to ward off an attack of impending gout. One observer experimented on himself, and found that when he lived on an exclusive vegetable diet he excreted 15 grains of uric acid per day, on a mixed diet 17 grains, and on an exclusive meat diet 21·5 grains daily.

